# Recent Advancements in Deep Learning Using Whole Slide Imaging for Cancer Prognosis

**DOI:** 10.3390/bioengineering10080897

**Published:** 2023-07-28

**Authors:** Minhyeok Lee

**Affiliations:** School of Electrical and Electronics Engineering, Chung-Ang University, Seoul 06974, Republic of Korea; mlee@cau.ac.kr

**Keywords:** whole slide images, cancer prognosis, survival analysis, image analysis, digital pathology, machine learning, artificial intelligence, medical imaging

## Abstract

This review furnishes an exhaustive analysis of the latest advancements in deep learning techniques applied to whole slide images (WSIs) in the context of cancer prognosis, focusing specifically on publications from 2019 through 2023. The swiftly maturing field of deep learning, in combination with the burgeoning availability of WSIs, manifests significant potential in revolutionizing the predictive modeling of cancer prognosis. In light of the swift evolution and profound complexity of the field, it is essential to systematically review contemporary methodologies and critically appraise their ramifications. This review elucidates the prevailing landscape of this intersection, cataloging major developments, evaluating their strengths and weaknesses, and providing discerning insights into prospective directions. In this paper, a comprehensive overview of the field aims to be presented, which can serve as a critical resource for researchers and clinicians, ultimately enhancing the quality of cancer care outcomes. This review’s findings accentuate the need for ongoing scrutiny of recent studies in this rapidly progressing field to discern patterns, understand breakthroughs, and navigate future research trajectories.

## 1. Introduction

The advancement of deep learning has incited a paradigm shift across a myriad of disciplines [1,2,3], notably within the medical sciences [4,5,6]. In the field of oncology, deep learning methods have showcased unparalleled capacities to extrapolate pertinent information from complex, high-dimensional data, thereby facilitating precise and timely diagnosis [7], treatment planning [8], and prognosis prediction [9]. In this context, whole slide images (WSIs) of cancerous tissues have surfaced as a crucial resource for prognosis prediction, attributing to their detailed and rich content that aptly depicts the disease’s multifaceted nature [10,11,12].

Deep learning has ascended as a potent computational paradigm by virtue of its capacity to model intricate hierarchical patterns in data [13,14]. It employs multilayered artificial neural networks to autonomously learn hierarchical representations from raw input data, thus considerably reducing the need for manual feature extraction. These representations, often termed as features, empower the model to distinguish and differentiate complex patterns in the data, rendering deep learning an apt tool for a multitude of tasks, encompassing image classification, natural language processing [15], and prognosis prediction [16], among others [17].

**Figure 1:**
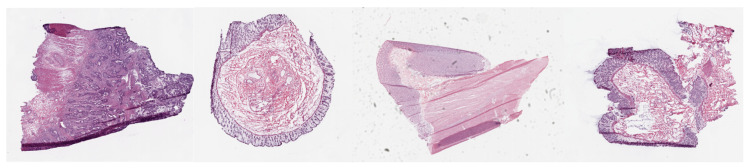
Illustrative whole slide images of colon adenocarcinoma in the TCGA-COAD dataset [21].

WSIs are digital slides derived from high-resolution scans of physical pathology slides, capturing detailed visual information about tissue structure and cellular morphology [18,19,20]. The high-resolution and multiscale nature of these images permit the representation of both the spatial context and the local texture within the tissue. This abundance of information makes WSIs a profoundly rich data source for deep learning models, enabling them to extract and learn complex patterns that may correlate with a patient’s prognosis. However, the substantial size and complexity of these images also pose unique computational and methodological challenges that necessitate skilled handling for effective utilization. A representative exemplification of WSIs is depicted in Figure 1.

The convergence of deep learning and WSIs represents an exciting avenue in cancer prognosis [22,23,24]. The dynamic interaction between the pattern discernment capabilities of deep learning algorithms and the voluminous, multiscale information inherent in WSIs facilitates an intricate depiction of the disease, potentially paving the way towards improved prognosis predictions. Despite its substantial potential, this intersection presents an array of challenges, not least of which include the necessity for substantial volumes of labeled data, the computational demands associated with processing high-resolution images, and the interpretability of deep learning models. It is imperative to address these challenges to successfully translate this technology into clinical practice, thereby offering a pathway towards more individualized and efficacious cancer treatment.

The complexity of cancers is closely entwined with the elaborate structural variations observable at the tissue level [25], and WSIs embody a rich source of information that captures this complexity across various scales. With the onset of digital pathology and increased accessibility of whole slide scanners, there has been a considerable surge in the availability of WSIs, thus providing a propitious environment for the application of advanced deep learning methodologies. Consequently, deep learning has been progressively incorporated into the pathology workflow, enhancing the human capacity for microscopic image analysis, furnishing prognostic predictions, and thereby offering a tangible route towards personalized cancer treatment.

While considerable progress has been achieved, the application of deep learning for cancer prognosis using WSIs is advancing at a remarkable pace. As the field progresses, it becomes indispensable to carry out a comprehensive review of contemporary developments. The rapid proliferation of the literature underscores the necessity for a prompt and exhaustive review that outlines recent advancements, discerns the strengths and limitations of current methodologies, and suggests avenues for future research. Thus, this review assumes critical importance, especially in the light of the fast-paced innovation characterizing the field.

This review paper will deliver a thorough exploration of deep learning applications for cancer prognosis utilizing WSIs, spanning a range of cancer types and meticulously cataloging cutting-edge models and methodologies. In the face of rapid advancement in deep learning techniques, it is vital to investigate the most recent research in order to identify trends, comprehend advancements, and envisage the future trajectory of this field. By offering a comprehensive summary of recent developments, this review aims to direct future research initiatives, inform clinical practices, and ultimately, facilitate the development of more efficient and personalized cancer treatments.

The accelerated advancements in deep learning have elicited growing interest in harnessing this technology for cancer prognosis using WSIs. Deep learning presents immense potential in revolutionizing the domain of cancer survival prediction through the provision of highly precise, efficient, and insightful computational methodologies. Hence, the principal objective of this scholarly paper is to present a thorough and meticulous review of recent literature deploying deep learning with WSIs for cancer prognosis, specifically focusing on publications from 2019 through 2023. In an epoch distinguished by unparalleled growth and innovation in deep learning, it is crucial for researchers to stay abreast of the latest developments in this dynamic field. This review serves as an invaluable resource for scholars, offering insights into cutting-edge techniques for analyzing WSIs utilizing deep learning methodologies, and contributing to the ongoing evolution of this discipline. By offering a comprehensive, current, and critical analysis, this paper aims to provoke thoughtful discussions, incite the conception of innovative ideas, and stimulate further advancements. Ultimately, these efforts strive to enhance outcomes for cancer patients by providing them with more accurate prognostic information.

The remainder of this paper unfolds as follows. In Section 2, a comprehensive analysis of the relevant literature is presented, covering the methodologies employed for paper selection (Section 2.1), a detailed examination of the selected studies (Section 2.2), an overview of the journals in which these studies were published (Section 2.3), the distribution of publication years (Section 2.4), and the citation distribution of these publications (Section 2.5). Subsequently, Section 3 delves into the specific application of deep learning techniques to whole slide images in studies focused on cancer prognosis. The findings and implications of these studies are then critically discussed in Section 4. Finally, Section 5 encapsulates the main conclusions of this review, illuminating the potential future trajectory of this promising field.

## 2. Literature Analysis

### 2.1. Methodology for Paper Selection

The fundamental goal of the paper selection process was to identify pertinent research studies that examine the application of deep learning methodologies on WSIs in the context of cancer prognosis. A carefully structured, algorithmic search strategy was employed, focusing primarily on the academic search engine, Web of Science (WOS). Three distinct search queries were utilized in this process: “Whole Slide Image Deep Learning Cancer Survival and Prognosis”, “Whole Slide Image Artificial Neural Network Cancer Survival and Prognosis”, and “Whole Slide Image Artificial Intelligence Cancer Survival and Prognosis”. These queries were combined using the Boolean operator ‘OR’, resulting in an initial set of 75 papers.

Subsequently, a manual curation of these studies was conducted to ensure relevance and alignment with the primary focus of “Deep Learning using WSIs for Cancer Prognosis”. Papers that did not maintain a direct link to cancer prognosis, such as those majorly focused on tumor segmentation, as well as review articles, were excluded from consideration. Studies employing artificial neural network models without deep architectures were also eliminated during this filtering process. Through this careful selection, papers that relied exclusively on traditional machine learning techniques without incorporating deep learning methodologies were excluded, thus refining the focus of the review on deep learning applications in cancer prognosis using WSIs. Consequently, 17 papers were removed from the initial pool, culminating in a final selection of 58 research papers for in-depth analysis in this review.

It is important to note that this review exclusively includes articles that have been published in peer-reviewed journals. This decision was driven by three main considerations. The peer-review process is an indispensable mechanism for ensuring research quality, subjecting it to stringent evaluation by experts in the field. Additionally, peer-reviewed scholarly journals are widely recognized as credible platforms for disseminating scientifically robust and impactful research. A further significant motive for exclusively incorporating articles from peer-reviewed journals in this review is the emphasis on scientific rigor and reliability. Peer-reviewed journals adhere to a meticulous process that involves independent experts critically appraising the research methodology, data analysis, and interpretation of results. This stringent evaluation aids in identifying and rectifying any potential shortcomings or biases in the research, thereby ensuring the integrity and credibility of the published work. By focusing on peer-reviewed articles, this review endeavors to offer a comprehensive and reliable synthesis of the current state of knowledge in the field of deep learning for cancer prognosis with WSIs. It enables readers to rely confidently on the findings and conclusions presented in the reviewed studies, aiding evidence-based decision making and fostering progress in the field.

Despite the abundance of preprints and conference papers in this field, a conscious decision was taken to focus solely on journal articles that have undergone peer review. The inclusion of this specific selection criterion serves to heighten the reliability and accuracy of the review, as it includes studies that have been subjected to rigorous evaluation. In order to preserve the novelty and originality of the review, certain categories of articles, such as review articles and perspectives, were purposefully excluded. This strategy seeks to highlight the inclusion of primary research-oriented studies, in line with the objective of the analysis.

The review was confined to articles published within a specific period of five years, namely from 2019 to 2023. The selected timeframe was deemed suitable to ensure the relevance and timeliness of the analysis, enabling a comprehensive understanding of the latest developments and trends in deep learning for cancer prognosis. It is pertinent to note that the data collection process for the year 2023 was concluded by May, to align with the existing timetable and keep the review current with the latest progress in the field. During the data collection phase, data pertaining to the citation count and publication history of each selected article were gathered. These specifics played a key role in assessing the extent, influence, and reception of the study within the academic community.

The papers that were chosen were subsequently classified based on the specific types of cancer that were the focus of each study. The aforementioned categorization enables a thorough comprehension of the deep learning landscape for cancer prognosis using WSIs by augmenting our comprehension of the varied methodologies utilized in this domain. The summary of the reviewed papers is presented in Table 1. Although it is recognized that numerous papers may overlap across various categories investigating multiple cancers, the approach was to assign them to a singular category, by making a category, namely multiple cancers, that is most relevant to the primary focus of the paper.

### 2.2. Analysis of Publications

Table 1 provides an overview of recent studies on deep learning with WSIs in cancer prognosis, categorized by cancer types. As observed, colorectal cancer is the most investigated cancer type, with twelve studies reported between 2019 and 2023. These studies, like those of Zhao et al. [26] and Sun et al. [28], utilized deep learning to analyze WSIs for colorectal cancer prognosis, focusing on different aspects like the structure of cancer cells and tissue morphology. Their work has led to more precise models capable of predicting cancer prognosis and progression.

Breast cancer is the second most investigated cancer type, with eleven studies. A notable example is Liu and Kurc [38], who used deep learning models to analyze WSIs and identify morphological patterns in breast cancer tissues. Their work has contributed to the development of highly accurate prognostic models, highlighting the capability of deep learning to handle complex and high-dimensional WSIs. Bladder cancer and liver cancer have also been areas of significant research, with eight and five studies, respectively. Zheng et al. [49] utilized deep learning with WSIs for bladder cancer prognosis, demonstrating the potential for more personalized treatments. For liver cancer, Saillard et al. [54] leveraged deep learning models to uncover intricate patterns in WSIs, contributing to improved understanding of the disease’s progression. Lung cancer and brain cancer have been the subject of five studies each. Pham et al. [59] and Shirazi et al. [63] used deep learning models to analyze WSIs, uncovering previously unidentified patterns and contributing to the development of highly accurate prognostic models.

There are also several studies focusing on less frequent cancer types like renal cell carcinoma, ovarian cancer, cervical cancer, gastric cancer, pancreatic cancer, skin cancer, head and neck cancer, bone cancer, and oral cancer. These investigations demonstrate the broad applicability of deep learning with WSIs across various cancer types, extending the potential benefits to a wider patient population. A category of studies have been dedicated to multiple cancers. For example, Shao et al. [80] used deep learning models to analyze WSIs from various cancer types. Their research underscores the potential of these models to generalize across different cancers and improve prognosis prediction.

This extensive survey of the discipline emphasizes the remarkable flexibility and applicability of deep learning approaches to WSIs in forecasting cancer prognosis. It simultaneously illuminates the imperative for persistent investigation across a diverse spectrum of cancer types. This is crucial to ensure that the advantages of this advanced technology are broadly accessible, fostering the evolution of more potent and tailored cancer therapies.

### 2.3. Publication Journals

The selected papers for this review have been published across a wide range of scientific journals, emphasizing the broad interest and applicability of deep learning with WSIs in the study of cancer prognosis. To provide an overview of the publication venues, a detailed and insightful analysis of these journals has been conducted.

The highest number of relevant papers were published in “Scientific Reports”, accounting for 10.3% of the total selection. Following this, “Cancers” and “Frontiers in Oncology” published a considerable number of pertinent papers, contributing 6.9% and 5.2% respectively. Other significant contributors include “Journal of Translational Medicine”, “American Journal of Pathology”, “Bioinformatics”, “IEEE Transactions on Medical Imaging”, and “Computer Methods and Programs in Biomedicine” (3.2%). However, more than half of the publications (60.4%) appeared in other journals with fewer than two publications each, thereby representing a considerable diversity in publication venues.

The diverse selection of journals in which the papers for this review were published highlights the interdisciplinary nature of deep learning with WSIs in cancer prognosis. Spanning across fields ranging from computer science to medical and biological research, the distribution of publications underscores the broad-ranging implications and wide acceptance of this methodology in the scientific community. By appearing in journals with different focuses, these papers contribute to the dissemination and integration of knowledge in multiple scientific domains, fostering collaboration and advancing the understanding of deep learning with WSIs in cancer prognosis.

### 2.4. Publication Years

Insights from the temporal distribution of the papers considered in this review reflect a rapid escalation in research interest and effort in the field of deep learning with WSIs in cancer prognosis. The chronological evaluation of publications elucidates the trajectory of this area of study and highlights the upsurge of research output over the last few years.

**Figure 2:**
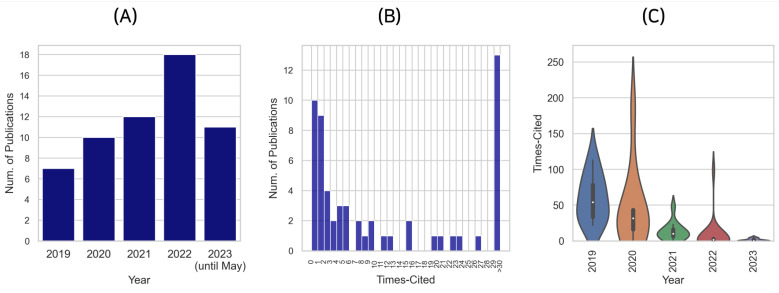
Distribution overview of publication years and citation frequencies. (**A**) presents the distribution pattern of publication years; (**B**) showcases the distribution of citation frequencies; and (**C**) depicts the relationship between the number of citations and the publication year of the papers.

As depicted in Figure 2A, the number of pertinent publications has experienced an upward trend from 2019 to 2022. The year 2019 saw the advent of 7 notable publications, which was surpassed by 10 publications in 2020, demonstrating an approximate 43% increase in scholarly output in a single year. The subsequent year, 2021, witnessed a modest rise, with 12 research contributions. However, a remarkable surge in the number of publications was observed in 2022, with a total of 18 relevant articles, marking a noteworthy 50% growth from the previous year. At the time of writing this review, the year 2023 (until May) had already accounted for 11 publications, indicating a sustained and thriving research interest in this area. If this trend persists, it is plausible to forecast that the total number of publications for 2023 will exceed those of previous years.

This increasing trend in the annual publication count is a clear testament to the escalating interest and recognition of the potential and relevance of deep learning with WSIs in the field of cancer prognosis. It also signifies the progressively growing academic response to the evolving challenges and opportunities presented by this exciting intersection of artificial intelligence and oncology. Such a trend encourages and propels further scientific inquiry, promising significant advancements in the pursuit of enhancing cancer prognosis using deep learning with WSIs.

### 2.5. Citation Distribution of the Publications

The examination of the citation distribution, in relation to the selected research papers, furnishes further context to comprehend the influence and reception of the academic work within this specific area of research. The citation count for a research paper typically serves as an indirect indicator of its impact and recognition within the scientific community.

As of the current collection period, the median citation count stands at 5, while the mean citation count has been computed to be 20.6. This discrepancy between the median and mean values can be attributed to a small number of highly cited papers that have influenced the average, signifying a skewed distribution, as illustrated in Figure 2B. This skewness can be understood better with the observation that a considerable number of studies have not been cited yet. One possible explanation for this pattern might be that these studies are relatively new, and thus, they have not had enough time to accrue citations. The citation count for 2023 has not been fully captured by WoS, further confirming this proposition.

Upon analyzing the relationship between the number of citations and the publication year, as represented in Figure 2C, it becomes evident that recent publications are yet to garner citations. More specifically, the median citation count of papers published in 2023 stands at zero. On the contrary, papers published in 2019 demonstrate a substantially higher impact, with a median citation count of 54. This distinction underlines the long-term influence and visibility of the research on deep learning with WSIs in cancer prognosis, affirming the substantial interest and potential growth of this research field in the years to come.

## 3. Deep Learning with Whole Slide Images in Studies on Cancer Prognosis

In the domain of cancer prognosis, significant strides have been made through the application of deep learning methodologies to WSIs. This approach has enabled researchers to develop predictive models for a wide range of cancer types. It is imperative to note that the interpretation of this rich and complex data has necessitated a myriad of sophisticated techniques, many of which have been adeptly crafted to fit the peculiarities of specific cancer types. The reviewed studies in this paper are summarized in Table 2.

Among the various prognostic models developed, several investigations have focused on specific cancer types. In brain cancer, Shirazi et al. [63] presented a deep convolutional neural network (CNN) called DeepSurvNet for survival predictions based on histopathological images. Deep learning models for survival prediction have also been developed for hepatocellular carcinoma (HCC). For instance, Saillard et al. [54] introduced SCHMOWDER and CHOWDER, while Hou et al. [57] proposed a multimodality prognostic model. Other studies, like those by Liu et al. [71] and Yokomizo et al. [70], focused on the prognosis of epithelial ovarian cancer (EOC) and ovarian clear-cell carcinoma (OCCC), respectively.

Attention to the tumor microenvironment (TME) is a common theme among several studies. Jiang et al. [48] and Jiao et al. [31] utilized CNNs to assess the TME in bladder cancer and colon adenocarcinoma, respectively. Liang et al. [55] introduced PathFinder, a deep learning framework that underscored the prognostic significance of the necrotic spatial distribution in liver cancer.

Several other studies have utilized CNNs for tumor–stroma ratio (TSR) quantification in various cancers. Zhao et al. [26] developed a deep learning model for TSR quantification in colorectal cancer (CRC). Similar models were also developed by Xu et al. [30] and Geessink et al. [36] for colorectal and rectal cancer, respectively.

Deep learning with WSIs has also been applied to quantify immune infiltration and cell distribution, with Yang et al. [29] introducing a deep learning-based metric called the Deep-immune score. In the domain of breast cancer, Fassler et al. [44] utilized machine learning and computer vision algorithms to characterize tumor-infiltrating lymphocytes (TILs), while Lu et al. [45] designed a deep learning-based pipeline to generate high-resolution TIL maps.

Another prominent direction is the integration of WSIs with clinical, genomic, or transcriptomic data. For instance, Liu et al. [65] developed cellular morphometric subtypes (CMS) using artificial intelligence for lower-grade gliomas (LGG), which independently predicted overall survival. Mao et al. [41] developed iCEMIGE, an integrative model combining cell morphometrics, microbiome, and gene biomarker signatures.

Several studies focused on the predictive power of pathological features in cancer prognosis. For instance, Chen et al. [73] designed a deep learning-driven pathological risk score to predict survival rates in cervical cancer. Similarly, Zhao et al. [27] formulated a deep learning methodology using the mucus–tumor ratio to assess colorectal cancer patient survival. An innovative approach by Levy-Jurgenson et al. [43] involved the use of deep learning models to spatially resolve gene expression levels in pathology WSIs, revealing significant correlations with survival rates.

The role of cellular composition, particularly TILs, in predicting cancer prognosis has been another significant research theme. Xu et al. [32] examined the prognostic impact of TIL spatial distribution in colorectal cancer, whereas Zheng et al. [49] used an artificial neural network classifier to identify tumor cells, lymphocytes, and stromal cells, underlining the prognostic value of electronic TIL variables in bladder cancer. Furthermore, Balkenhol et al. [40] utilized a convolutional neural network to investigate the role of TILs in the prognosis of triple-negative breast cancer. Several studies have placed emphasis on the prognostic value of the tumor microenvironment, and specifically TILs. Gavriel et al. [51] employed machine learning for predicting a 5-year prognosis in muscle-invasive bladder cancer (MIBC), integrating image, clinical, and spatial features. Shaban et al. [79] introduced a method to quantify TIL abundance in oral squamous cell carcinoma histology images, proposing the TILAb score as a potent prognostic indicator for disease-free survival.

Exploring multimodal features in WSIs for prognostic predictions has also attracted research attention. Chen et al. [64] proposed pathomic fusion, an end-to-end multimodal fusion strategy for predicting survival outcomes in cancer patients. Cheerla and Gevaert [81] also developed a multimodal neural network-based model for pancancer prognosis prediction. Similarly, Wu et al. [53] conducted a multimodal analysis of bladder cancer data, combining CT scans, WSIs, and transcriptomics, which led to a convolutional neural network for molecular subtyping with increased accuracy. Zhang et al. [77] introduced a prognostic nomogram integrating microscopic digital pathology and macroscopic magnetic resonance images for predicting survival in nasopharyngeal carcinoma.

Focusing on specific cancer types, deep learning methods have been deployed for early-stage lung adenocarcinoma prognosis (Shim et al. [60]), ovarian cancer prognosis (Wu et al. [72]), and recurrence-free survival prediction in hepatocellular carcinoma (Qu et al. [56]). Additional efforts include a CNN-based system for identifying gastric mucosa abnormalities (Ma et al. [74]), and the use of CNNs for classifying renal cell carcinoma subtypes (Tabibu et al. [67]).

The potential of weakly supervised learning models has also been explored, with Shao et al. [80] proposing BDOCOX, a weakly supervised deep ordinal Cox model for survival prediction from WSIs. In similar vein, Zheng et al. [50] developed weakly supervised deep learning models for diagnosing bladder cancer and predicting overall survival rates. Shi et al. [58] proposed a weakly supervised deep learning framework for hepatocellular carcinoma analysis, establishing a ’tumor risk score’ from WSIs that outperformed traditional clinical staging systems in predictive ability.

Furthermore, advanced techniques like multiresolution deep learning methods have been employed for survival analysis in breast cancer (Liu and Kurc [38]), and multihead attention mechanisms have been used for survival prediction using WSIs (Jiang et al. [83]). On another note, a two-step deep learning algorithm was suggested to improve the detection of lung cancer lymph node metastases from WSIs (Pham et al. [59]).

Several studies have delved into the potential of pathological features as potent prognostic indicators. For instance, Sun et al. [28] engineered a prognostic signature for colorectal cancer using deep learning techniques, while Xu et al. [33] introduced WDRNet, a dual-resolution network that demonstrated promising capabilities for both colorectal cancer diagnosis and prognosis. Focusing on colorectal cancer from a different perspective, Xu et al. [34] constructed an AI model to quantify the Crohn’s-like lymphoid reaction (CLR) and TILs, creating a CLR-I score as a significant prognostic indicator for overall survival.

A considerable amount of research has been dedicated to the development of advanced deep learning models for prognosis prediction in specific types of cancer. Jaber et al. [39] developed a deep learning model to approximate PAM50 intrinsic subtyping in breast cancer, and Knuutila et al. [76] employed residual neural network architectures to differentiate metastatic tumors from primary nonmetastatic and metastatic cutaneous squamous cell carcinomas (cSCCs). Marostica et al. [68] designed a fully automated CNN for diagnosing renal cancers and predicting survival outcomes. Liu et al. [69] proposed a two-step framework for prognostic prediction using WSIs and tumor mutation burden (TMB) in clear-cell renal cell carcinoma (ccRCC).

One notable area of focus is the prediction of metastasis and recurrence risk in diverse types of cancer. For instance, Klimov et al. [75] formulated a deep learning pipeline for assessing metastasis risk in pancreatic neuroendocrine tumors (PanNET). With a focus on lung cancer, Guo et al. [61] applied deep learning to the analysis of immune checkpoint staining, enabling the prediction of survival and relapse in non-small-cell lung cancer. Complementarily, Wang et al. [62] conducted a multicenter study that designed a pathology image texture signature from WSIs. This signature demonstrated significant prognostic value and improved survival discrimination when combined with clinicopathological variables for lung adenocarcinoma.

In the study of colorectal cancer, Chen et al. [35] proposed a fully automated risk stratification approach based on gland formation, employing a deep survival model for enhanced prediction. Meanwhile, Shapcott, Hewitt, and Rajpoot [37] utilized a deep learning cell identification algorithm on colon cancer WSIs. Their technique predicted morphological features connected to cellularity and uncovered substantial associations with clinical variables such as metastasis.

Breast cancer studies have also leveraged deep learning and machine learning methods. Wang et al. [42] developed an MIL fusion model that merged pathological images and clinical features to predict the prognostic risk of HER2-positive breast cancer patients. In another innovative approach, du Terrail et al. [46] combined WSIs and clinical data to predict the histological response to neoadjuvant chemotherapy in triple-negative breast cancer, employing federated learning to secure data privacy and improve performance. Adding to this body of work, Xu et al. [47] carried out an integrated analysis of histopathological images and chromatin accessibility data in estrogen receptor-positive breast cancer. They used deep learning for tissue segmentation and canonical correlation analysis to unearth regulatory regions linked to prognosis.

Deep learning techniques have also made considerable inroads in the study of other cancer types. For instance, Jiang et al. [66] designed deep learning models for prognosis and IDH mutation status prediction in grade 2 gliomas. Ho et al. [78] devised a deep learning network for assessing the necrosis ratio in osteosarcoma WSIs. This innovative technique allows for objective and reproducible evaluations and aids in patient stratification for survival prediction.

## 4. Discussion

In the recent literature, a variety of deep learning models have been deployed to analyze WSI data within the prognosis of oncology contexts. CNNs have emerged as a prevalent choice due to their effectiveness in handling image data, demonstrated by studies such as those conducted by Wu et al. [53], Xu et al. [34], and Zhang et al. [77]. A considerable number of investigations have also employed variations of CNN, often categorized under the umbrella term of general deep learning. This group includes diverse studies by Liang et al. [55], Zheng et al. [49], and Jiang et al. [48]. Residual networks, a form of CNN, have been frequently implemented, as seen in works by Liu et al. [69], Wu et al. [72], and Knuutila et al. [76], who utilized the ResNet model due to its ability to effectively train very deep neural networks. Of late, there has been an uptick in the application of attention mechanisms, which allocate varying levels of importance to different parts of the image. For instance, Jiang et al. [83] employed a multihead attention mechanism and Wu et al. [72] combined ResNet with attention mechanisms. Such models have been increasingly favored due to their capability to focus on crucial regions of an image while simultaneously considering the context, thus improving their interpretability and prediction accuracy. Yet, as the field rapidly expands, it becomes increasingly clear that numerous challenges persist that necessitate ongoing research and innovation.

A primary challenge is the variance in data quality, which is an issue pervasive across all medical imaging analyses [48,70]. A broad range of factors including staining inconsistencies, scanning variations, and differences in slide preparation methodologies can induce a significant level of noise in the data, posing difficulties for the robustness of deep learning models. For example, inconsistencies in H&E staining protocols have been reported to impede the reproducibility of results and to compromise the performance of models trained on different datasets [58]. Future work in this field could focus on developing techniques for mitigating these data quality issues, such as stain normalization methods or robust feature extraction techniques.

Another challenge lies in the interpretability of the deep learning models [36,45]. The black-box nature of these models can be an impediment to their acceptance in the medical community, where understanding the decision-making process can be as important as the decision itself. While progress has been made towards developing interpretable deep learning methods [73], future work could further focus on elucidating the underlying decision mechanisms of these models. Specifically, developing techniques for visualizing feature importance, deciphering hidden layer activations, and understanding model predictions could be areas of interest.

The heterogeneity of cancer types also poses significant challenges to the development of generalizable models [38,72]. Different cancer types are characterized by distinct histopathological features, and these variations can complicate the application of models trained on one type of cancer to another. Despite some promising initial steps towards developing multicancer models [64,81], more research is required in this area. One potential approach could be to focus on developing methods for transfer learning, enabling models trained on one type of cancer to adapt to others.

The integration of multimodal data, such as combining WSIs with genomic, transcriptomic, or clinical data, has been shown to enhance predictive power [41,65]. However, such integration poses its own challenges, including data harmonization, dealing with missing data, and developing models that can effectively learn from different data types. Future work could focus on developing novel methodologies for the seamless integration of multimodal data, thus enabling more accurate and comprehensive cancer prognosis predictions.

One promising direction for future research is the exploration of weakly supervised learning models for cancer prognosis. Such models, as proposed by Shao et al. [80], Zheng et al. [50], and Shi et al. [58], could potentially alleviate the reliance on extensive manual annotations, reducing the workload of pathologists and accelerating the development of predictive models. Moreover, the use of advanced techniques like multiresolution deep learning methods and attention mechanisms, as employed by Liu and Kurc [38] and Jiang et al. [83], should continue to be investigated for more sophisticated analysis and more accurate prediction results.

In addition to the challenges mentioned above, the computational resources required to train deep learning models on WSIs constitute another hurdle [38,41]. Due to the high-resolution nature of WSIs, the size of these images can be enormous, leading to increased memory requirements and extended processing times. Although hardware acceleration techniques and parallel computing have been employed to alleviate this issue, the development of more efficient models and optimization algorithms should be explored in future work.

Also, there remains the challenge of validating and deploying these models in real-world clinical settings [63,80]. While the studies covered in this review demonstrate promising results in research environments, it is crucial to assess how these models perform in practice, with the heterogeneous and noisy data typically encountered in clinical scenarios. This validation process would involve meticulous evaluation across diverse patient populations and multiple clinical sites. Future work should therefore place a stronger emphasis on such external validation studies to bridge the gap between research and practice.

Furthermore, there are ethical and legal considerations in the deployment of AI models in healthcare, which cannot be overlooked [34]. Issues related to data privacy, informed consent, and potential biases in AI models need to be adequately addressed. Guidelines and regulations need to be established to ensure the responsible and ethical use of AI in cancer prognosis.

Looking ahead, there are several promising avenues for future work. The application of novel AI technologies, such as multitask deep learning models [84], to WSI analysis could lead to more powerful and flexible models. The exploration of unsupervised and self-supervised learning methods could provide ways to leverage the large amounts of unlabeled WSIs, potentially uncovering novel histopathological features predictive of cancer prognosis.

Additionally, developing robust and interpretable models that can adapt to changes over time, such as the evolution of cancer morphology or treatment effects, could offer exciting prospects. In line with this, there is a growing interest in integrating deep learning models with dynamic temporal data, such as longitudinal clinical data or time-series molecular data, to capture the temporal aspects of cancer progression. Ultimately, the goal is to advance the field in such a way that deep learning models can serve as reliable and useful tools for pathologists, augmenting their capabilities, reducing their workload, and contributing to more accurate and timely cancer prognosis, ultimately improving patient outcomes.

## 5. Conclusions

Based on the comprehensive literature review presented, it is evident that the application of deep learning techniques on WSIs has brought remarkable advancements in the field of cancer prognosis. These novel methodologies have not only enabled the processing of large amounts of complex histopathological data, but they have also facilitated the development of sophisticated predictive models, enhancing the accuracy and reliability of cancer prognosis.

The research reviewed demonstrates a broad spectrum of applications across various types of cancer, each with its distinct characteristics and complexities. From leveraging CNNs for survival predictions in brain and liver cancer to exploring tumor microenvironments in bladder and colon cancers, these methodologies have made significant strides in enhancing our understanding of cancer prognosis. The innovative integration of WSIs with clinical, genomic, or transcriptomic data points to the potential for a truly holistic approach to cancer prognosis.

The quantification of immune infiltration and cell distribution through the application of deep learning models further underscores the richness of the insights that can be derived from WSIs. These advancements in predictive modeling have not only improved our understanding of various cancer types but also facilitated the development of effective strategies for diagnosis, treatment, and management.

The significant progress made in the field of cancer prognosis using WSIs is encouraging, indicating a promising future for cancer diagnosis and management. The ability to accurately predict survival rates and recurrence risk using deep learning methods has significant implications for clinical practice and patient care. As more sophisticated models and techniques are developed, the potential to revolutionize the field of oncology is immense.

While the accomplishments made thus far are substantial, the field is still in its infancy, with considerable scope for further research and development. Challenges persist, including the need for more comprehensive, diverse, and robust datasets, the development of standards for the evaluation of predictive models, and the need for integrating these advanced techniques into clinical practice. As the field matures and these challenges are addressed, the potential for deep learning techniques applied to WSIs to transform the field of cancer prognosis is immense.

The advancements made in the field of deep learning applied to WSIs for cancer prognosis are promising. They offer the potential for a significantly improved understanding of the complex interplay of factors influencing cancer prognosis, and the development of more accurate and personalized strategies for cancer management. The future of this field is vibrant with possibilities, and the continued exploration and refinement of these methodologies will undoubtedly contribute to significant improvements in cancer prognosis and patient care.

## Figures and Tables

**Table 1:** Overview of deep learning with whole slide images (WSIs) in studies on cancer prognosis, categorized by cancer types.

Cancer Type	Studies
Colorectal Cancer	Zhao et al. [26], Zhao et al. [27], Sun et al. [28], Yang et al. [29], Xu et al. [30], Jiao et al. [31], Xu et al. [32], Xu et al. [33], Xu et al. [34], Chen et al. [35], Geessink et al. [36], Shapcott, Hewitt, and Rajpoot [37]
Breast Cancer	Liu and Kurc [38], Jaber et al. [39], Balkenhol et al. [40], Mao et al. [41], Wang et al. [42], Levy-Jurgenson et al. [43], Fassler et al. [44], Lu et al. [45], du Terrail et al. [46], Xu et al. [47]
Bladder Cancer	Jiang et al. [48], Zheng et al. [49], Zheng et al. [50], Gavriel et al. [51], Brieu et al. [52], Wu et al. [53]
Liver Cancer	Saillard et al. [54], Liang et al. [55], Qu et al. [56], Hou et al. [57], Shi et al. [58]
Lung Cancer	Pham et al. [59], Shim et al. [60], Guo et al. [61], Wang et al. [62]
Brain Cancer	Shirazi et al. [63], Chen et al. [64], Liu et al. [65], Jiang et al. [66]
Renal Cell Carcinoma	Tabibu et al. [67], Marostica et al. [68], Liu et al. [69]
Ovarian Cancer	Yokomizo et al. [70], Liu et al. [71], Wu et al. [72]
Cervical Cancer	Chen et al. [73]
Gastric Cancer	Ma et al. [74]
Pancreatic Cancer	Klimov et al. [75]
Skin Cancer	Knuutila et al. [76]
Head and Neck Cancer	Zhang et al. [77]
Bone Cancer	Ho et al. [78]
Oral Cancer	Shaban et al. [79]
Multiple Cancers	Shao et al. [80], Cheerla and Gevaert [81], Fu et al. [82], Jiang et al. [83]

**Table 2:** Used deep learning methods, strengths, and limitations of the reviewed studies.

Ref.	Deep Learning Methods	Expected Strengths	Expected Limitations
[83]	Multihead Attention (Attention Mechanisms)	Comprehensive WSI analysis outperforms existing approaches and contributes to prognosis prediction.	Not specified
[55]	General Deep Learning (including MLP)	Potential biomarkers discovered provide enhanced prognostic performance.	Interpretability and generalizability limitations may hinder clinical acceptance.
[69]	ResNet	Cost-effective tumor mutation burden measurement and prognostic biomarkers outperform original TMB signature.	Not specified
[78]	Deep Multimagnification Network	Highly correlated necrosis ratio estimation and outcome prediction.	Dependence on manual review of necrosis ratio from multiple slides.
[46]	Federated Learning	Privacy-preserving multicentric studies with interpretable ML model.	Biases from small-scale study and time-consuming expert annotations.
[53]	CNN	Potential for multimodal data use in clinical applications with high diagnostic accuracy.	Not specified
[72]	ResNet, Attention Mechanisms	Risk stratification facilitated in ovarian cancer through deep learning framework.	Moderate mean value of C-index; uneven prediction strength across subgroups.
[42]	Multiple-Instance Learning (MIL), GAT, Attention Mechanisms	Novel MIL fusion model enables accurate prognostic risk prediction.	Not specified, potential challenges with image segmentation and representation.
[62]	ResNet-50	MPIS integration with clinicopathological variables improves LUAD prognostic stratification.	Transferability of MPIS to all cancer types uncertain.
[50]	Weakly Supervised Deep Learning	Accurate bladder cancer diagnosis and personalized treatment decisions.	Not specified
[49]	General Deep Learning (including MLP)	The proposed model improves survival prediction in bladder cancer by assessing TILs.	Not specified
[71]	CNN, Attention Mechanisms	High-performance prognosis prediction in Epithelial ovarian cancer using AI mechanisms.	Not specified
[33]	General Deep Learning (including MLP)	High-accuracy colorectal cancer prognosis using a weakly supervised deep learning network.	Not specified
[29]	General Deep Learning (including MLP)	Deep learning-based immune index correlates strongly with colorectal cancer survival rates.	Not specified
[57]	General Deep Learning (including MLP)	Multimodality prognostic model provides high-performance survival prediction in hepatocellular carcinoma.	Not specified
[48]	General Deep Learning (including MLP)	Depiction of tumor microenvironment immunophenotypes offers insights into biological pathways in bladder cancer.	Not specified
[41]	Sparse Representation Learning	The proposed model improves risk stratification in breast cancer with integrated biomarkers.	Effectiveness tied to biomarker extraction quality; untested outside of breast cancer.
[73]	CNN with Autoencoder	Deep learning-based pathological risk score predicts cervical cancer prognosis.	Prediction performance tied to dataset quality; clinical application untested.
[65]	Autoencoder with Regularization	CMS discovery allows personalized diagnosis in lower-grade gliomas.	Limitations with validating subtypes for other cancer types and accounting for inter-tumor heterogeneity.
[76]	ResNet	The proposed model identifies morphological features associated with metastasis in cSCC.	Performance tied to data quality and diversity; untested outside of cSCC.
[38]	Deep Learning with Multiresolution	Deep learning method for breast cancer survival integrates image data, improving model performance.	Needs more validation; performance varies with data quality.
[28]	Variational Autoencoder (VAE), Generative Adversarial Network (GAN)	Improved prognostic signature for stratifying outcomes in stage III CRC.	Limited generalizability to other cancer types or stages.
[35]	Spatial Pyramid Network	Automated CRC risk stratification approach related to gland formation.	Model may require further refinement despite better discrimination.
[44]	ResNet-34	TIL infiltrates assessment in breast cancer WSIs acts as significant biomarkers.	Dependence on TIL infiltrates; performance in TIL absence unclear.
[32]	General Deep Learning (including MLP)	Prognostic utility for CRC PFS prediction based on automatic TIL quantification.	Performance tied to TIL quantification; unclear performance in TIL absence.
[64]	General Deep Learning (including MLP)	End-to-end multimodal fusion improves survival outcome prediction.	Performance tied to availability of paired WSI, genotype, and transcriptome data.
[34]	CNN	The proposed model for CLR and TIL quantification improves survival prediction in CRC.	Needs further validation on larger cohorts for generalizability and clinical deployment.
[70]	ResNet-34	The proposed model achieves high accuracy for prognosis in OCCC.	Single-institution data may limit model generalizability.
[80]	General Deep Learning (including MLP)	The proposed model reduces interoperator variation in survival prediction from WSIs.	Efficiency compromised by WSI size and pattern heterogeneity.
[30]	CNN	Stroma-immune score using deep learning improves survival prediction in CRC.	Larger validation cohorts needed for reliable assessment of model’s prognostic value.
[66]	ResNet-18	Improved prognosis and IDH mutation status prediction in lower-grade gliomas.	Small sample size may limit robustness and generalizability.
[60]	CNN	The proposed model utilizes multiscale pathology images for prognosis prediction in lung adenocarcinoma.	Not specified
[61]	EfficientUnet, ResNet	Efficient analysis of immune checkpoints and prognosis of NSCLC.	Not specified
[68]	CNN	Accurate RCC subtype diagnosis and prediction of survival outcomes.	Interrater variability and limitations in capturing all biological signals.
[58]	Weakly Supervised Deep Learning	Prognostic indicators from HCC pathological images improve risk stratification.	Efficiency and labor-saving limitations; needs further validation for patient treatment.
[51]	Ensemble Learning	Prediction of MIBC prognosis significantly higher than TNM staging system.	Further validation and clinical deployment needed.
[40]	CNN	Efficient assessment of TILs in triple negative breast cancer provides valuable prognostic information.	Optimal prognostic information yielding method unclear; lack of objective TIL assessment methods.
[75]	CNN	High accuracy in predicting metastasis risk in pancreatic neuroendocrine tumors.	Not specified
[27]	General Deep Learning (including MLP)	Accurate mucus proportion quantification in colorectal cancer suitable for clinical application.	Not specified
[47]	General Deep Learning (including MLP)	Integrative analysis of histopathological images and genomic data improves understanding of disease progression.	Might not identify all potential regulatory regions in the human genome.
[54]	General Deep Learning (including MLP)	Two deep learning algorithms aid risk stratification for hepatocellular carcinoma patients.	Not specified
[77]	Convolutional Neural Networks (CNN)	Prognostic model predicts treatment failure in nasopharyngeal carcinoma better than existing clinical models.	Not specified
[43]	General Deep Learning (including MLP)	The models developed can spatially characterize tumor heterogeneity. Showed a significant statistical link between heterogeneity and survival.	Lack of automated methods for characterizing tumor heterogeneity.
[26]	CNN, Transfer Learning	Automated deep learning method for TSR quantification in colorectal cancer reduces pathologist workload.	Not specified
[74]	CNN	CNN-based system distinguishes tissue types with high accuracy in gastric diseases.	Not specified
[82]	Transfer Learning	Deep transfer learning quantifies histopathological patterns across a range of cancer types.	Not specified
[45]	CNN	High-resolution TIL map generation on WSIs strongly associates with immune response pathways and genes.	Not specified
[63]	CNN	Exceptional accuracy in brain cancer survival rate classification based on histopathological images.	Challenges in generalizability on unseen samples and practical clinical application.
[39]	General Deep Learning (including MLP)	Deep learning classifier identifies breast cancer molecular subtypes and heterogeneity.	Potential inaccuracies due to intratumoral heterogeneity.
[59]	General Deep Learning (including MLP)	Two-step deep learning approach accurately detects lung cancer metastases.	Presence of false positives in model predictions.
[79]	General Deep Learning (including MLP)	TILAb score predicts disease-free survival in OSCC patients better than manual TIL score.	Accuracy tied to quality and clarity of WSIs.
[67]	Convolutional Neural Networks (CNN)	High accuracy distinguishing renal cell carcinoma subtypes and predicting patient survival.	Class imbalance issues in medical datasets.
[81]	Multimodal Neural Network	Model combining clinical, mRNA, microRNA data, and WSIs predicts survival for 20 cancer types.	Not specified; potential complexity in interpreting multiple data modalities.
[36]	General Deep Learning (including MLP)	Automated approach determines TSR as an independent prognosticator in rectal cancer.	Applicable only in user-provided stroma hot-spots; performance tied to input image quality.
[37]	General Deep Learning (including MLP)	Deep learning algorithm for cell identification in colon cancer images improves performance.	Patch selection for analysis may impact results.
[52]	CNN	Quantification of tumor buds in bladder cancer adds prognostic value to traditional TNM staging.	Not specified
[56]	CNN	Recurrence-related histological score allows for clinical decision making in HCC recurrence prediction.	Prediction accuracy varies; potential bias towards trained data and diseases.
[31]	CNN	Automatic evaluation of the tumor microenvironment in WSIs aids in predicting disease progression.	Varied strength of predictors; potential bias towards specific cancer types.

## Data Availability

No new data were created or analyzed in this study.
